# Mouse Models for Efficacy Testing of Agents against Radiation Carcinogenesis—A Literature Review

**DOI:** 10.3390/ijerph10010107

**Published:** 2012-12-27

**Authors:** Leena Rivina, Robert Schiestl

**Affiliations:** 1 Department of Environmental Health Sciences, University of California, Los Angeles, 650 Charles E. Young Dr. South, CHS 71-295, Los Angeles, CA 90095, USA; 2 JCCC Healthy and At-Risk Populations Program Area, Department of Pathology and Laboratory Medicine, University of California, Los Angeles, 650 Charles E. Young Dr. South, CHS 71-295, Los Angeles, CA 90095, USA; E-Mail: rschiestl@mednet.ucla.edu; 3 Department of Radiation Oncology, University of California, Los Angeles, 650 Charles E. Young Dr. South, CHS 71-295, Los Angeles, CA 90095, USA

**Keywords:** radiation carcinogenesis, animal models, radiation protectors, radiation mitigators, secondary cancers

## Abstract

As the number of cancer survivors treated with radiation as a part of their therapy regimen is constantly increasing, so is concern about radiation-induced cancers. This increases the need for therapeutic and mitigating agents against secondary neoplasias. Development and efficacy testing of these agents requires not only extensive *in vitro* assessment, but also a set of reliable animal models of radiation-induced carcinogenesis. The laboratory mouse (*Mus musculus*) remains one of the best animal model systems for cancer research due to its molecular and physiological similarities to man, small size, ease of breeding in captivity and a fully sequenced genome. This work reviews relevant *M. musculus* inbred and F_1_ hybrid animal models and methodologies of induction of radiation-induced leukemia, thymic lymphoma, breast, and lung cancer in these models. Where available, the associated molecular pathologies are also included.

## 1. Introduction

The number of people diagnosed with cancer each year is growing, as is the number of post-therapy survival rates. Approximately one in two people in the United States will be diagnosed with cancer at some point in their lifetime and about half of them will receive radiation as a part of their therapy regimen [1,2]. Radiation is either administered as a sole curative/palliative agent, in combination with chemotherapeutic drugs, molecular targeted therapy, immunotherapy, or as a part of immune suppression procedure for bone marrow, stem cell and organ transplantation [3]. However, normal healthy tissues are inadvertently exposed to radiation, which may result in a variety of acute toxicities or chronic secondary malignancies. One of such malignancies is radiation-induced cancer [4,5].

In recent years, rapid technological advances in radiation oncology have enabled radiation to be targeted much more precisely to tumor sites reducing some unnecessary exposure of healthy surrounding tissues and increasing both the maximum tolerated doses and the therapeutic ratio [6,7]. However, collateral exposure of normal tissue and potential subsequent malignancy is still unavoidable. Development of biological therapies to supplement technological advances in radiation oncology would present a powerful solution and may further revolutionize the field.

There are three potential classes of agents intended to modulate normal tissue damage: (1) radiation protectors, agents given prior to radiation exposure; (2) radiation mitigators, agents given post-exposure (PE) but prior to the onset of symptoms; and (3) therapies, or agents administered after the onset of symptoms [8]. To date, Amifostine™ is only agent approved by the Food and Drug Administration (FDA) intended to protect normal tissues during irradiation [9]. To increase the number of available radiation modulating therapies, National Cancer Institute (NCI) in collaboration with the National Institute of Allergy and Infectious Diseases (NIAID) have put forth an algorithm for preclinical and clinical development for agents aimed at decreasing the adverse effects of cancer therapy, including radiation [10]. One of the imperative parts of the proposed algorithm is accurate selection of animal models for therapeutic activity validation. For in-depth review on the selection of NIAD-recommended animal models for the testing of therapies designed to mitigate or treat non-cancer toxicities associated with radiation exposures one can refer to Williams *et al.* [11]. The purpose of this work is to review select inbred mouse models that may be used in preclinical settings to test the efficacy of agents intended to protect, mitigate, or treat radiation-induced carcinogenesis.

## 2. Methods

### 2.1. Research Strategy

*Mus musculus*, the laboratory mouse, is one of the best models available for the study of cancer initiation, progression and corresponding pathologies. The laboratory mouse has undergone a significant evolution in its complexity enabling it to mimic more and more precise aspects of the most multifaceted disease of all—cancer. In the researcher’s arsenal today are murine models ranging from carcinogen-inducible tumors to xenograft models transplanted with human neoplastic cells to humanized mice that express human genes. New generations of genetically engineered mice (GEM) have been imbued with the ability to accurately recapitulate pathophysiological and underlining molecular features of many human cancers [12]. Genetically homogenous inbred mice used in environmentally inducible cancer studies are increasingly neglected in favor of GEMs, often because the inbred mice develop tumors at low frequencies and with variable latencies. Despite these flaws, however, inbred mice are indispensible in the discovery of novel oncogenes, tumor suppressors and preclinical assessment of toxic or therapeutic effects of innumerable agents [13].

We have set out to identify inbred mouse models of radiation-induced (RI) cancers intended for assessment of efficacy in protecting, mitigating or treating these malignancies. We have concentrated on models of leukemia, lung and breast cancers, as these have been identified as the most commonly arising secondary cancers post radiation therapy [5]. Lymphoma has also been included, despite its unconfirmed status as a radiation-induced malignancy in man.

### 2.2. Inclusion Criteria

The scope of this review is limited to murine models of radiation-induced leukemogenesis, lymphomagenesis, breast and lung carcinogenesis following exposure to low-LET gamma- and X-ray radiations with a high total dose and dose-rate. Inductions with high-LET radiation, genetically engineered mice and models that required supplemental treatment are outside of the scope of this work. Only inbred mice whose cancers are inducible with either a single total body irradiation (TBI) or fractionated targeted exposures are described, the only exception being an orthograft radiation chimera model of breast cancer. Lastly, only murine models mimicking underlying molecular pathologies observed in man are included.

## 3. Results and Discussion

### 3.1. Radiation-Induced Leukemia

Leukemia was one of the few cancers recognized as a radiation-induced malignancy quite early in the development of the field of radiation biology. Before any radiation safety standards were introduced, many X-ray workers, mostly physicists and engineers, developed leukemia after working near accelerators and other ionizing radiation (IR) sources. However, much more than anecdotal correlation between radiation exposure and increased leukemia incidence and mortality began emerging as the reports from Life Span Study cohorts following Japanese atom-bomb survivors and patients receiving high doses of therapeutic radiation for cervical cancers, tinea capitis and ankylosing spondylitis began to be published [5,14,15,16,17,18,19]. In a large study conducted by Boice and colleagues, the risk of secondary malignancies following radiation treatment for the uterine cervix carcinoma established as a sharp increase in leukemia incidence following irradiation [20]. In the last decades, more reliable data from the Chernobyl disaster on excess risk estimates of leukemia in adults and children also began to emerge, providing a more complete data set on age-dependence, doses and latencies [21,22,23,24].

Despite the differences in exposure scenarios, irradiation dose-rates and doses, and radiation quality components, there are salient features common to all reported IR-induced leukemias. Acute and chronic myeloid leukemia (AML and CML, respectively) are the two most common radiation-induced cancers observed in the adult human population [15,16,18,25,26,27]. Children exposed at 5–9 years of age appear to be more susceptible to acute lymphocytic leukemia (ALL), while older children are more likely to develop AML. Chronic lymphocytic leukemia (CLL) does not seem to be influenced by radiation [14]. The risk of developing leukemia is highest within the first decade following exposure and begins to decrease as time goes on, but never quite returns to baseline risk [15,16,21,26,28]. In some reports, sex differences have been reported in radiation-induced leukemia instances [16,18,21,25].

Epidemiological data, however, cannot tell the whole story about radiation-induced leukemogenesis. The use of mouse models becomes imperative to study the mechanism of induction, improve diagnostics, and further radiation protection, therapy and mitigation efforts. Today a few established IR-induced leukemogenesis murine models exist: RF [29,30], SJL/J [31], CBA [32,33], and C3H/He [34]. Table 1 summarizes the optimal methods of induction and the associated myeloid leukemia (ML) frequencies.

**Table 1:** Induction of myeloid leukemia in mice with low-LET ionizing radiation.

Malignancy	Mouse Strain	Age	Sex	Mode of Induction	Latency	Spontaneous Frequency	Induced Frequency	Ref.
Myeloid Leukemia	RF (RF/J, RFM)	8 weeks	Male	4.25 Gy	4–12 months	2–4%	50–90%	[30]
Myeloid Leukemia	SJL/J	8–10 weeks	Female	3–3.5 Gy	12 months	0 %	10–30%	[31]
Myeloid Leukemia	C3H/He	8–10 weeks	Male	2.84 Gy	1.5–18 months	<1%	25%	[34]
Myeloid Leukemia	CBA (CBA/Ca, CBA/Cne, CBA/H)	12–15 weeks	Male	3 Gy	18–24 months	<1%	~25%	[32,33]

#### 3.1.1. RF Mouse

The RF mouse was developed at the Rockefeller Institute as a general-purpose stock from A, R, and S strains [30,35,36]. Its propensity for radiation-induced leukemogenesis has been extensively studied by Upton and colleagues [37]. One of the earliest accounts of leukemogenesis in these mice dates back to 1936 with detonation experiments conducted by Furth and colleagues [38]. Myelogenous or myeloid leukemia (ML) in the RF model is inducible with a single dose of ionizing radiation and has been proposed as a valid counterpart to human AML, particularly due to its prolonged preclinical period with diagnosable pre-cancerous tissue lesions [30]. 

Background incidence of myeloid leukemia in RF mice is 2–4% and appears later in life, at around 18–24 months of age [39]. Exposure of 8-week old RF males to 1.5 Gy increases ML frequency to about 40% while *in utero* and neonatal exposures actually decrease ML induction [29,40]. At the dose of 4.25 Gy ML incidence increases to 50–90%, with a latency period of 4–12 months [30,37,41]. As early as 12 weeks post exposure, an enlarged spleen and liver accumulate young myeloid cells. Clinically, RF leukemia presents with infiltration to peribronchial areas, lymph nodes, and gastrointestinal lymphoid organs. However, at the same dose the induction of thymic lymphoma also increases to about 25%, which can potentially interfere with accurate ML diagnosis and modeling of the human disease [30]. Upton *et al.* have also demonstrated a sex difference in susceptibility to TL and ML: females are more susceptible to TL, while males are more likely to develop ML [29].

Hayata *et al.* reported that, similarly to radiation-induced leukemia in the SJL/J mouse [42], myeloid leukemia in the RF model exhibits partial deletion of chromosome #2 along with other genomic instabilities including loss of the Y-chromosome [43]. Some researchers have suggested that the protracted latency of ML in RF mice correlates with the data from Japanese A-bomb survivors and children exposed in the Chernobyl disaster, with the corresponding peak incidence in leukemia 5–10 years following the irradiation event [16,21,25,44]. One drawback of the RF mouse model is the fact that it often presents with mixed hematopoietic tumors of myeloid leukemia and thymic lymphoma [29].

#### 3.1.2. SJL/J Mouse

Developed in the 1960s by Murphy, the SJL/J strain is known for its high spontaneous frequency of reticulum cell neoplasms (type B, RCN B) [45,46] that occur at roughly 380 days of age in both males and females. The histological pattern observed in the RCN B was similar to that of Hodgkin’s disease in human beings, leading to the proposal for its use as an investigative model of such lesions [47].

Single exposure of 8–10-week old female SJL/J mice to 3.0–3.5 Gy of whole-body irradiation induces myeloid leukemia in 10–30% of treated animals within a year. However, Haran-Ghera and Kotler have also observed that SJL/J exposure to fractionated X-rays induces lymphosarcomas [47]. Consistent with AML diagnosis, leukemic infiltrations are observed in the bone marrow, lymph nodes, spleen and liver [31]. The frequency of radiation-induced acute myeloid leukemia (RI-AML) increases with age up to about 12 weeks during the time of irradiation. Such an increase is possibly explained by the sensitivity of the developing mononuclear phagocytic system [48].

While radiation appears to initiate the progression of RI-AML, this multiphase malignancy often requires additional promoting factors for tumor development [49]. Preleukemic cells and the characteristic chromosome 2 deletions are observed in the overwhelming majority of bone marrows of IR-treated mice prior to overt AML clinical presentation (90–120 days) [50,51]. Additionally, administration of corticosteroids following irradiation increases RI-AML incidence to 50–70% [31]. Administration of growth factors, especially colony stimulating factor-1 (CSF-1), decreases latency and increases frequency to 75% [49,52]. In fact, 2–4 months prior to RI-AML onset, preleukemic RJL/L mice have significantly elevated CSF-1 levels as compared to mice that fail to develop RI-AML or those that develop RCN B. RI-AML cells *in vitro* synthesize significant amounts of CSF-1, further supporting CFS-1 necessity for leukemia progression in addition to IR [48].

Clinical presentation of RI-AML in SJL/J mouse resembles that of secondary leukemias observed in man [31]. Patients in remission after radiation and steroid treatment for Hodgkin’s disease often develop AML similar to those described in SJL/J mice [53,54,55]. Additionally, elevated circulating levels of CSF-1 have been reported in neoplastic malignancies including AML and appear to be associated with poor prognosis [56,57,58,59], further supporting the use of the SJL/J mouse for the study of CSF-1’s role in cancer.

#### 3.1.3. C3H Mouse

Strong developed the C3H strain in 1920 from a cross of the Bragg Albino mouse and the DBA mouse, specifically selecting for elevated incidence of mammary tumors (MT). Ninety percent of unfostered pups (pups remaining with mother postpartum) develop mammary tumors by 11 months of age. Fostering the offspring or transferring fertilized ova to a mammary tumor virus-free surrogate significantly reduces tumor frequency [35,36]. Fostered C3H/He substrain has a high incidence of spontaneous hepatomas later in life [34,60]. 

X-irradiation of 8–10 week old male C3H/He mice with 3 Gy TBI induces myeloid leukemia in 23.9% of exposed animals, with myelomonocytic leukemia being the most prevalent subtype. Dose-response curves for C3H mice are similar to those for RFM and CBA mice: there is a proportional increase in the frequency of leukemia induction until a critical dose of around 3 Gy, after which the incidence spontaneously drops off [32]. Spontaneous incidence of leukemia is less than 1% [34].

Yoshida *et al.* reported a significant sex difference, with females being less susceptible to RI-ML than males of the same age. Administration of the synthetic glucocorticoid prednisolone following irradiation of C3H/He mice increases the incidence of ML to 38.5% in a similar fashion to that of SJL/J mice [31]. The mechanism of induction is suspected to involve suppression and promotion of hematopoietic recovery. Reducing daily caloric intake by roughly a third eliminates spontaneous ML entirely and decreased the incidence of RI-ML to 7.9% when the restriction started before 6 weeks of age or to 10.7% when the restriction started post exposure (PE) at 10 weeks of age [61]. Caloric restriction also promoted PE longevity via insulin pathway modulation [62]. Chronic inflammation may also be implicated as an exacerbating and possibly a leukemogenesis-promoting factor. In later studies Yoshida demonstrated that inducing chronic low-level inflammation by inserting a cellulose acetate membrane increases RI-ML incidence to 35.9% [63].

As with the RFM and SJL/J mice, partial deletion of chromosome 2 has been implicated in RI-AML in C3H/He mice [43,64]. As early as 24 h PE, during the first metaphase, chromosome 2 deletions are detectable in the bone marrow in the C3H/He mouse, suggesting that chromosome 2 deletions act in the initiation stages of leukemogenesis [65]. Some researchers have compared human Ph^1^ chromosome transformations in chronic myeloid leukemia to mouse chromosome 2 aberrations in its incidence and disease specificity [66,67].

#### 3.1.4. CBA Mouse

The CBA mouse is a cross between a Bragg albino female and a DBA male originally developed by Strong in 1920 with low mammary tumor incidence. Males of CBA/Ca substrain tend to have a shorter lifespan than CBA/Ca females [35,36]. Both CBA/Ca and CBA/H are direct substrains of the original CBA mouse derived in the United Kingdom [68,69].

A 3.0 Gy TBI irradiation with either gamma- or X-rays of 12-week old male CBA/H mice results in 25% induction of myeloid leukemia. Infiltration of the sternal bone marrow, liver, and spleen are observed and serve as diagnostic endpoints [32,33]. As previously mentioned, the dose-response curve is curvilinear, implying a threshold dose similar to that of man—leukemia is rarely observed in cases with high exposure [70,71].

As with the other mouse models of RI-ML, chromosome 2 aberrations have been reported and correlated with myeloid leukemia in the CBA mouse [69,72,73]. From as early as 20 hours to as late as 24 months PE, expansion of cells carrying chr2 lesions is observed in 20–25% of irradiated mice [74]. Bouffler *et al.*, however, weren’t able to conclude that the induction of chr2 aberrations and presence of an aberrant chr2 clone can accurately predict development of RI-AML in CBA mice [75]. Aberrations on chromosome 4 in about 50% of CBA/H mice diagnosed with typical AML were also reported. Cleary *et al.* have identified the *Lyr2*/TLSR5 allele as a likely mutation candidate for radiation-induced hematopoietic malignancies including the myeloid and lymphoid mouse leukemias [76]. Susceptibility to RI-AML in CBA/H has also been linked to an 8% decrease in DNA-methylation not observed in the AML-resistant strain C57Bl/6 [77].

The CBA mouse is the current favorite RI-AML model for human AML for a few reasons: (1) it has a low spontaneous frequency of AML, (2) it has a favorable mean latency of 18 months, and (3) morphologically CBA AML resembles the human malignancy [68,78]. Dekkers *et al.* have also suggested that the two-step mutation model of RI-AML in CBA/H, extrapolated from X-ray and neutron data, has application in animal modeling of human RI-AML [79].

#### 3.1.5. ML-Associated Molecular Pathologies

For over 30 years, deletions on chromosome 2 have been linked to AML in murine models across multiple strains (RF, C3H/He, CBA, and SJL/J) [42,43,64]. Due to the specific nature of the chromosome aberrations on chr2, the loss of a tumor suppressor gene seemed a more likely scenario than an oncogene activation [80]. In 2004 Cook and colleagues identified the *sfpi1* gene encoding a transcription factor PU.1 from the 2 Mbp commonly deleted region on chr2 [80,81,82].

PU.1/*Sfpi-1* is an important player in hematopoiesis and is involved in promotion, differentiation and regulation of all hematopoietic lineages. It’s essential for terminal myeloid (macrophages and neutrophils) cell differentiation and stem cell maintenance [83,84,85,86,87]. In mice, PU.1 function is important for leukemic transformations in myeloid cells; in humans its importance in such transformations is still actively debated [81,88,89]. PU.1 has a DNA binding domain, engages in protein-protein interactions and has regulatory phosphorylation sites imperative for its function [90].

In addition to the loss of PU.1 on one chr2, the second copy of PU.1 is often inactivated by point mutations in the DNA binding region [81,88]. Homozygous conditional knockdown of PU.1 (expressing ~20% of WT levels) induces AML in mice by 3–8 months of age [91] and myeloid leukemia when inactivated in adult mice [92]. In transgenic mice expressing oncoprotein PML-PAR, loss of genomic region coding for PU.1 is a common secondary event in leukemogenesis [93]. Upregulation of *c-Myc* has also been reported in AML cells accompanying PU.1 deficiencies [94]. Cook *et al.* have demonstrated that expression of PU.1 at WT levels in promyelocytic leukemia cells inhibited clonogenic growth, forced monocytic differentiation, and induced apoptosis. All of these findings suggest that suboptimal expression of PU.1 can provoke and promote leukemogenesis by blocking maturation of the cell [81,87]. Peng *et al.* have also suggested that quantification of PU.1-deleted bone marrow cells may be used as a surrogate marker for RI-AML [95].

In humans a homologue of PU.1 exists on chromosome 11 [87] and is expressed at low levels in most AML cases [96]. However, direct inactivation by deletion of PU.1 is very rare [88,89]. Cook proposes that other mechanism of PU.1 inactivation in human AML might be at play: the gene might be silenced epigenetically, through protein-protein interaction or via interaction with a mutated receptor (*i.e.*, Flt3 cytokine receptor that are found in 25% of human AML) [81]. Interestingly, Finnon *et al.* have recently shown that *Flt3*-ITD and *Sfpi*1/PU.1 mutations are mutually exclusive in murine radiation-induced AML without any overt phenotypic differences [97]. The group has not reported actual levels of PU.1 in these RI-AMLs, so it is plausible that the PU.1 depression is still involved in these malignancies.

It remains unclear, however, whether radiation is responsible for one or both genomic events observed in RI-AML: deletion of PU.1 on chr2 and *Sfpi*1 mutations in its DNA-binding domain. Data suggests that IR induces chr2 deletions [51,64,95], but it remains undetermined whether the deletion is a result of direct DNA damage or induced through delayed genomic instability [98,99,100]. Radiation, however, is not a likely candidate for the direct alteration of the second PU.1 allele in RI-AML cells, as IR does not induce point mutations observed in *Sfpi*1 [81,88,94]. Point mutations are the most common type of spontaneous mutations and evidence suggest that *Sfpi*1 mutations are of spontaneous origin [101,102].

Ban and Kai demonstrated that replicative stress applied to hematopoietic stem cells (HSC) surviving 3 Gy radiation contributes to the HSC’s accelerated aging, thus decreasing replicative fidelity of the genome and increasing the rate of mutation accumulation, including mutations in the remaining copy of the *Sfpi*1 gene. A mathematical model fitted to experimental data from cobblestone area forming cells (CAFC) and colony forming unit-granulocyte/macrophages (CFU-G/M) on *ex vivo* bone marrows revealed that irradiated HSCs cycle as much as ten times more quickly than those from unexposed animals [102]. Such increase in cycling is thought to also appear *in vivo* after irradiation. 

Hirouchi *et al.* have recently challenged the commonly accepted paradigm that the HSC is the targeT cell of RI-AML [78] and concluded that AML stem cells can arise from long-lived HSCs as well as the short-lived multipotent progenitors (MPPs) and common myeloid progenitors (CMPs) that have acquired self-renewal potential. The cell surface phenotypes and gene expression profiles of AML stem cells in the study closely resembled those of normal CMPs rather than those of HSCs [103].

In addition to chr2, critical loci on chromosomes 8, 13, and 18 have also been identified. On chr18 resides *Rbbp8* gene that encodes for the CtIP protein. CtIP is upregulated in response to X-ray exposure in the RI-AML-sensitive CBA mice but not in the RI-AML-resistant C57BL/6 and is a suspected tumor suppressor. Aberrant human chromosome segments bearing *Rbbp8* gene have been reported in many cancers including AML [104].

### 3.2. Radiation-Induced Lymphoma

Debate is still ongoing regarding the causal effect of ionizing radiation on lymphomagenesis in man. While Hartge and colleagues concluded that IR probably causes lymphoma and a small increase in risk between radiotherapy and lymphoma has been identified [105,106], a plethora of investigators tend to disagree. Some investigators found the link between non-Hodgkin’s lymphoma (NHL) and radiotherapy extremely weak, and no association at all between IR and Hodgkin’s disease [16,107,108,109]. Recent data, however, seem to suggest that the causal link is real. Richardson *et al.* have published data supporting a strong link between ionizing radiation and lymphoma mortality among radiation workers exposed at the Savannah River Site in South Carolina. The basis for disagreement among researchers appears to be the disease’s protracted latency and obscure mechanism of induction [110].

Historically, in rodent pathology classification no distinction was made between lymphomas and lymphocytic leukemias. Malignant lymphomas in mice can be placed into six subdivisions with further modifiers depending on the site of the tumor—thymic, mesenteric, and leukemic [111]. Most of the time murine lymphoma is diagnosed phenotypically: labored breathing, hunched posture, and the enlargement of spleen and lymph nodes are indications of fulminant malignancy. Mice with labored breathing but without enlarged spleens and lymph nodes are usually classified as thymic lymphomas [112]. Immunological markers and morphologic criteria are also commonly used in more specific diagnosis [113,114,115]. Immunophenotypes of thymic lymphoma in mice closely resemble their counterparts in humans despite the fact that there is no direct human analog of thymic lymphoma [115].

Thymic lymphoma (TL) in mice has been extensively studied as a model of radiation-induced carcinogenesis since it was first described by Kaplan *et al.* in 1953 [116]. In addition to C57BL/6 and other C57BL substrains, BALB/c and NSF are also susceptible to RI-TL [117,118] (See Table 2 for summary).

**Table 2:** Induction of thymic lymphoma in mice with low-LET ionizing radiation.

Malignancy	Mouse Strain	Age	Sex	Mode of Induction	Latency	Spontaneous Frequency	Induced Frequency	Ref.
Thymic Lymphoma	C57BL (C57BL/6, C57BL/6J)	4–6 weeks	Male, Female	4 fractions of~1.7 Gy once a week	3–6 months	<1%	>90%	[116,119,120]
Thymic Lymphoma	BALB/c (BALB/cHeA)	4 weeks	Male, Female	4 exposures ~1.7 Gy once a week for 4 weeks	2.5–9.5 months	5–6% females; 0% males	77 % (Females) 86% (Males)	[112,117]
Thymic Lymphoma	NFS	4 weeks	Male, Female	4 fractions ~1.7 Gy once a week for 4 weeks	3–6 months	>1% within 12 months	90% (females) 89% (males)	[121,122]

#### 3.2.1. C57BL Mouse

C57BL mice were developed in 1921 as a cross between female 57 and male 52 from Miss Abbie Lanthrop stock. It is one of the most widely used mouse stocks in the laboratory. Up to 7% of C57BL/6 mice develop spontaneous leukemia [36,123].

As early as 1949, Sacher and Brues were able to induce thymic lymphoma in mice with X-ray radiation [124]. In 1952 Kaplan *et al.* published a seminal paper identifying optimal fractionation periods for TL induction at 8-day intervals for 4 weeks in C57BL mice that results in 93% disease penetrance within ~250 days following the first irradiation. Females were identified to be slightly but significantly more susceptible than males to TL at 58% *versus* 47%, respectively. Lungs and peripheral lymph nodes seem to be affected in the majority of murine lymphoma cases [119]. TL with similar frequencies and latency periods can be induced in C57BL/6, C57BL/10 and C57BL/Ka substrains of C57BL [120,125,126].

Radiation-induced thymic lymphoma (RI-TL) is highly asynchronous and lymphoma cells have been often staged by the presence of MEL-14^hi ^(lymphocyte homing receptor), H-2K^hi ^(histocompatibility antigen), and IL-2R^+ ^(interleukin 2 receptor) surface markers on thymus cortical cells. In the normal adult thymus less than 3% of the cells in the cortex express MEL-14^hi ^or IL-2R^+ ^[127,128] and the presence of MEL-14^hi ^may signify appearance of a leukemic clone [129]. Additionally, most of the TL tumors bear T-lymphocyte specific antigens Thy-1, Lyt-1, and Lyt-2 [130,131].

The most detected early chromosome abnormality observed in IR-induced thymic lymphoma in C57BL mice is trisomy of chromosome 15, detected in 65–71% of case [132,133]. Chromosome 15 trisomy is one of the most common cytogenic abnormalities in murine cancers as it leads to amplification of the oncogene *myc,* deregulation of which might be important in TL [134,135]. Alteration of *myc* expression through a translocation is observed in nearly all Burkitt’s lymphoma (BL) cases in man. *Myc* is an oncogene and a transcriptional factor regulating apoptosis; its deregulation has been observed in many cancers in addition to BL [136]. Activation of N*-ras* and *K-ras* has also been reported in just over 50% of RI-TL case in C57BL/6J [128,137]. Inactivation of tumor suppressor *p53* does not seem to be a salient feature of RI-TL in C57BL/6 mice [135] but transgenic *p53* knockout mice do exhibit higher frequency of RI-TL and implicate another tumor suppressor *Pten* [138].

#### 3.2.2. BALB/c Mouse

BALB/c is an inbred strain acquired by Bagg in 1913 and further expanded by Snell in 1932, who has subsequently added the /c designation to reflect the “color” homozygous color locus. It is among one of the most commonly used strains and purportedly does not develop lymphatic leukemia, but is sensitive to radiation lethality [36].

Following the Kaplan *et al.* methodology, thymic lymphoma can be induced in BALB/c mice with fractionated radiation (1.7 Gy/fraction, four fractions total) beginning at 4 weeks of age in both male and female mice at 86% and 77%, respectively. Later in life, females exhibit a spontaneous frequency of lymphoma at 5.5% but males do not. The mean latency for both sexes is ~5 months after IR [112,117]. 

The majority of studies on the mechanism of lymphomagenesis have been historically worked out either in C57BL/6 and its substrains or in hybrids that have included a BALB/c parent mated with a strain resistant to radiation-induced lymphoma. Recently, however, the inbred BALB/c mice have been used to demonstrate the role of microRNA (miRNA) in radiation-induced lymphomagenesis. Liu *et al.* concluded that in RI-TL tissues tumor suppressor gene *Big-h3* is downregulated while miR-21 is upregulated. MiR-21 is likely to directly target Big-h3 by inhibiting translation in a 3' UTR dependent manner [139]. 3' UTR dependent manner assumes a specific binding of miRNA to mRNA targets in the 3' untranslated region (3' UTR) [140].

#### 3.2.3. NFS Mouse

NFS is an inbred strain derived from the outbred NIH Swiss-Webster introduced to Japan in 1972. Maintained in sister-brother mating its current designation is NFS or NIH Swiss/S. The strain is also available in the United States [118].

Thymic lymphoma in NFS mice is induced in the same fashion as in BALB/c and C57BL mice: 4 weekly irradiations of 1.7 Gy beginning at one month of age. Both males and females are susceptible with comparable frequencies but the latency in males is longer (167 *vs.* 208 days). Spontaneous frequency is also low at less than 10% at 600 days of age [118]. Thymectomy on pre-irradiated NSF animals reduces the incidence of TL but increases the incidence of nonthymic lymphomas and leukemias in 67% of treated mice. Nonthymic lymphomas were predominantly observed in the spleen and mesentery lymph nodes and were most likely of B-cell origin [121]. Perhaps, thymectomies prior to irradiation might provide for a more relevant model of human lymphoma.

#### 3.2.4. TL-Associated Molecular Pathologies

From the 1980s on the use of hybrid models in radiation-induced thymic lymphoma studies became more common as it allowed for easier detection of underlying molecular pathologies. Popular hybrids included (C57BL/6J × BALB/c)F_1_, B6C3F1 (C57BL/6J × C3H)F_1_, C3B6F1 (C3H × C57BL/6)F_1_, (BALB/c × MSM)F_1 _[122], (C57BL/6J × RF/J)F_1 _[141], (C57BL/6J × DBA)F_1_, CBA/H × C57BL/6 [142,143] and the CXS series of recombinant inbred strains derived from TL-susceptible BALB/cHeA and TL-resistant STS/A [112]. The frequencies of RI-TL in the hybrids between highly susceptible strains and those with low susceptibilities (e.g., BALB/c × MSM) are usually between the expected frequencies of the parental strains. However, at times the hybrid mice require higher radiation doses in the four weekly fractions, *i.e.*, 2.5 Gy instead of the usual 1.7 Gy [122]. The use of these hybrids has elucidated the importance of such molecular players as *Ikaros/Znf1a1* [144,145], *BCL11B*/*Rit1* [146], *p73* [147], *p19/ARF* [148], and inactivation of *p15/INK4b(Cdkn2b)* and *p16/INK4a(Cdkn2a)* [149] in radiation-induced lymphomagenesis. *Ikaros/Znf1a1* protein is encoded by the *Ikzf1* gene and has many functions including immune system development and regulation of hematopoietic differentiation. In recent years downregulation of *Ikaros* activity has been established as the most clinically relevant tumor suppressor in B-cell acute lymphoblastic leukemia (B-ALL) and its downregulation associated with poor prognosis [150]. *BCL11B*/*Rit1* (B-cell CLL/lymphoma 11b) belongs to the largest family of transcription factors, the Kruppel-like C2H2 type zinc finger transcription proteins, and is involved in T-cell differentiation. BCL11B is also a tumor suppressor and is reportedly downregulated in 10–16% of T-cell acute lymphoblastic leukemias (T-ALL) [151,152]. *p73* is a member of the *p53*, pro-apoptotic tumor suppressor family [153]. Low levels of *p73* mRNA have been reported in non-Hodgkin’s lymphoma (NHL) but not in reactive hyperplasia patients. *p73* inactivation in NHL cases appears to be due to aberrant methylation of its promoter [154]. Inactivation of cyclin dependent kinase inhibitors from the INK4 family (ARF, p15, and p16) have been reported in Non-Hodgkin’s and Burkitt’s lymphoma cases [155,156]. Table 3 summarizes the most relevant molecular pathologies observed in radiation-induced thymic lymphoma in mice.

**Table 3:** Relevant molecular pathologies in murine RI leukemia and lymphoma.

Mouse Strain	Malignancy	Molecular Pathology	Role in Cancer	Ref.
RF; SJL/J; C3H/He; CBA	Myeloid Leukemia	Chr2 deletions: loss of *PU.1/Sfpi1* on one chr2 copy; inactivation on the second copy	Oncogene and transcriptional regulator of myeloid promoters PU.1 suppression linked to leukemic transformation in mice and men	[80,81,82,88,89]
C57BL	Thymic Lymphoma	–Trisomy of chr15: *myc* implicated	–Oncogene and transcription regulator of many cell events including apoptosis —Almost ubiquitous deregulation in Burkitt’s lymphoma	[131,132,133,134]
Hybrids between C57BL/6, C3H, BALB/c, MSM, and RF/J, CBA, DBA, and the CTX	Thymic Lymphoma	Loss of *Ikaros/Znf1a1* activity	Gene expression regulation and chromatin remodeling in hematopoietic differentiation One of the most clinically-relevant tumor suppressors in acute lymphoblastic leukemia (B-ALL)	[144,145] [121,150]
		Loss of *BCL11B*/*Rit1* activity	Transcription factor and tumor suppressor Linked to T-cell acute lymphoblastic leukemia (T-ALL)	[146,151,152]
		Loss of *p73* activity	*p53* family tumor suppressor Abrogated expression in non-Hodgkin’s lymphoma	[147,153,154]
		Loss of *p19/ARF, p15/INK4b* (*Cdkn2b*) and *p16/INK4a* (*Cdkn2a*) activity	Cyclin dependent kinase inhibitors that restricT cell cycle progression at G_1_ Non-Hodgkin’s and Burkitt’s lymphomas	[142,148,149,155,156]

Transgenic expression of activated *Notch1* in murine lymphocytes induces lymphomagenesis [157]. Activation of *Notch1* and inactivation of *Notch2,* paired with overexpression of *c-Myc* and defective *Znfn1a1/Ikaros* has been reported in 81.25% of RI-TL suggesting their molecular collaboration in lymphomagenesis [158]. Additionally, the use of hybrids has elucidated recurring chromosomal aberrations on chromosomes 4, 11 and 12, but these aberrations do not appear ubiquitously in all hybrids (e.g., BALB/C × MSM does not show LOH on chr 4 but C57BL/6J × RF/J does) [143]. Saito *et al.* have reported a specific susceptibility locus near *D4Mit12* on chromosome 4, as well as loci at *D2Mit15* (chr2) and *D5Mit15* (chr5). [122]. Piskorowska and colleagues identified additional susceptibility loci at a sex-dependent locus on chr10 (*D10Mit134*), and chr12 (*D12Mit52I*) [159].

Hybrids between C57BL/6 and C3H (C3B6F1 and B6C3F) share similar aberrations resulting in copy-number reduction and allelic loss at *Ikaros* and *Bcl1b* but not at the *Cdkn2a/Cdk2b* and *Pten* when compared to their parental strains. Interestingly, *Ikaros* and *Bcl1b* alterations are due to multilocus deletions, while *Cdkn2a/Cdk2b* and *Pten* show uniparental disomy. In these specific mice, *Ikaros* appears to be lost first followed by *Bcl11l* at a later time, in contrast with BALB/c × MSM hybrids where the order is reversed [132]. Rearrangements within the T-cell receptor alpha, *Tcra,* are also more common in these C57BL/6 and C3H hybrids as compared to those of the T-cell receptor beta, *Tcrb*, although both aberrations are observed. Allelic loss of *Tcrb,* a more strictly regulated allele, suggests that increased aberrant V(D)J rearrangement or increases in illegitimate V(D)J recombination might be important in IR-induced lymphomagenesis and may be the basis for strain differences in susceptibility to RI-TL [132]. Deficiencies in V(D)J activity have also been associated with intragenic deletion in *BCL11B* and *Notch1* in human lymphoid malignancies [160,161,162].

Kominami and Niwa have expanded the idea of an indirect mechanism of RI-TL, where IR may contribute to induction through genomic instability, but does not necessarily target thymocytes for the promotion of TL [163]. Fractionated total body irradiation leads to thymocyte apoptosis and that in turn leads to differentiation arrest and population regeneration by the damaged but surviving thymocytes with tumorigenic potential [124,164,165,166]. Transplantation or intravenous infusion of unirradiated bone marrow into an irradiated host reduces lymphomagenesis possibly by restoring the thymic microenvironment and preventing clonal expansion of irradiated T-cell precursors. Similarly, bone marrow shielding protects against RI-TL possibly through the same thymus repopulation mechanism. Inversely, transplantation of a non-irradiated thymus into an irradiated animal can develop into full TL [165,167,168,169]. Additionally, Muto and colleagues demonstrated that intrathymic (i.t.) and intraperitoneal (i.p.) injections of thymocytes from irradiated donors 4 months post-IR into unirradiated hosts results in T-type lymphomas of the donor type. At one month post-IR only intrathymic injections result in donor-type lymphomas in the recipient host suggesting the necessity of the thymus for further promotion in these “prelymphoma cells.” Identical experiments but with bone marrow cell injections did not induce lymphomas in recipients indicating that the bone marrow might not be the site of origin of the prelymphoma cells [170]. Furthermore, RF mice subjected to thymectomy prior to irradiation have a reduced incidence of TL down to 1% from 32% [29]. Collectively, all of these findings suggest that IR targets additional cells and tissues, not only the thymocytes, at the origin of this malignancy and that the thymic environment plays an important role in TL promotion. 

Notch ligands and receptors appear to at least partially mediate the interaction between thymus tissues and hematopoietic progenitors leading to lymphoma [163]. Further contributing to the indirect model of lymphomagenesis might be alterations in regulation of reactive oxygen species (ROS) in surviving thymocytes, leading to the accumulation of ROS-induced mutations [171,172]. Tamura *et al.* have identified a candidate susceptibility gene *Mtf-1* (metal responsive transcription factor-1) and later demonstrated that a certain variant of *MTF-1*, found in susceptible BALB/c mice, is linked to more proliferating premature thymocytes with higher ROS levels than in the strain of mice resistant to RI-TL [173,174]. *MTF-1* is activated by heavy metals and is involved in post-radiation signaling pathways regulating intracellular ROS [175].

While thymic lymphoma is a malignancy observed in mice but not in man, radiation-induced lymphomagenesis models can offer important insight into the progression of hematopoietic neoplasias in humans as well. *Ikaros,* identified in RI-TL mice, has also been implicated in human acute lymphoblastic leukemia (ALL), the most common hematopoietic malignancy among children [144,176,177,178,179]. Tsuji *et al.* demonstrated the contribution of illegitimate V(D)J recombination to *Notch1* 5'-deletions in radiation-induced thymic lymphoma, deregulation of which is thought to be involved in etiology of B- and T-cell human lymphomagenesis [180]. *Notch1*, a diverse master regulator responsible for a plethora of cellular processes, is itself an important player in both RI-TL and T-cell acute lymphoblastic leukemia [181]. Similarly, *PTEN* [182] and *CDKN2A/CDKN2B* have been proposed as candidates for initiation and/or progression of human ALL [183,184]. A new target gene, *EPHA7*, has been recently uncovered in the RI-TL mouse model and correlated with human T-cell lymphoblastic leukemia/lymphoma (T-LBL). EPHA7 is inactivated in 100% of T-LBL in mice and 95.23% of humans by either loss of heterozygocity, promoter hypermethylation or a combination of both [185].

### 3.3. Radiation-Induced Lung Cancer

In most industrialized nations, including the United States, lung carcinoma accounts for about a quarter of all cancer deaths, with the majority of cases being attributable to tobacco smoke [186]. Of the cancers associated with radiation, lung carcinoma was one of the first to be identified due to its high mortality [187]. Historically, data on radiation’s contribution to lung carcinogenesis has primarily come from three groups of exposed individuals: (1) underground miners exposed to alpha radiation through radon-222 and radon-220 inhalation, (2) patients treated with IR for neoplastic and non-neoplastic malignacies, (3) and the Japanese atomic bomb survivors [4,188,189]. Minimal latency for gamma and X-ray exposed patients appears to be 9–10 years with a persistent increase in risk remaining over 25 years after exposure. Females tend to be considerably more susceptible to radiation-induced lung cancer than men when researchers have accounted for the confounding factor of smoking. Based on the data from Japanese survivors, adenocarcinoma appears to be the most common type of lung cancer in the exposed population, and no correlation is apparent between age at time of exposure and malignancy risk [4,190,191]. Travis and colleagues have reported a significant increase in all histopathological types of lung cancer in Hodgkin’s disease patients treated with radiation up to 40 Gy or more after controlling for smoking. The incidence of secondary lung cancers in Hodgkin’s patients peaks at 5–9 years following radiation therapy [192,193,194]; the reason for this shorter latency remains to be established. Recently, the data set has been complemented by new studies reporting an increase in lung cancer incidence in women treated with radiation for breast cancer [195,196].

In both humans and animals, delayed effects of radiation exposure are pulmonary fibrosis, the replacement of normal tissue with connective tissue fibers, and carcinogenesis. The induction of pulmonary fibrosis *versus* the induction of lung cancer appears to be a function of dose, with carcinogenesis requiring a much smaller dose [187]. A report by Williams *et al.* provides an extensive guide to animal model selection for radiation fibrosis in addition to other radiation-induced malignancies [11]. This review summarizes three models of radiation-induced lung cancer employing whole body irradiations and targeted thoracic exposures in C3H, BALB/c and RF mice. Table 4 summarizes the strains and methodologies of induction.

**Table 4:** Induction of lung cancer in mice with low-LET ionizing radiation.

Malignancy	Mouse Strain	Age	Sex	Mode of Induction	Latency	Spontaneous Frequency	Induced Frequency	Ref.
Lung Cancer	C3H (C3H/HeSlc)	6 weeks	Male	2 fractions of 7.5 Gy to the thorax 12 h apart	12 months	3.5–9.5%	40%	[190,191]
Lung Cancer	RFM (RFM/Un)	10–12 weeks	Female	9 Gy to thorax	9 months	~28%	87%	[193,194]
Lung Cancer	BALB/c (BALB/c/An)	12 weeks	Female	2 Gy TBI	12 months	~12%	~37%	[195,196]

#### 3.3.1. C3H Mouse

C3H/He mice have a low spontaneous frequency of lung tumors and moderate sensitivity to radiation-induced lung tumorigenicity [197]. While the highest frequency of induction at ~62% is observed with a 7.5 Gy thorax irradiation followed by three 3 Gy whole body irradiations at 3-month intervals, it is potentially irrelevant to lung radiation carcinogenesis in humans. A more clinically relevant scenario is a two-fraction irradiation at 7.5 Gy with a 12-hour interval between the irradiations. This exposure scenario results in a ~40% induction in males irradiated at 6-weeks of age. After a 12-month latency, treated mice tend to develop alveologenic adenomas and adenocarcinomas; tubular- or papillary-form tumors are rarely observed [198]. In a series of dose-response studies, Hashimoto and colleagues showed that tumor incidence following a single WBI increases up to 5.0 Gy and then begins to decrease, supporting a previously suggested model of competitive dynamics between inductive and suppressive effects of radiation [199].

Night irradiations are much more potent inducers of RI-LT in C3H mice than exposures during the day. To achieve the same tumor frequency as that seen at night with a 1.25 Gy irradiation a 5 Gy irradiation is required during the day [198]. Diurnal variations have been also reported in responses to cancer therapy in man as well and have been the basis for a clinical study [200]. C3H appears to be the optimal choice for ionizing radiation-induced lung carcinogenesis with its low spontaneous and moderate induction frequencies. Coggle and colleagues suggested that induction with thoracic irradiations is the preferred method [187] because it is clinically more relevant and reduces the incidence of other tumors in the animal that can contribute to lethality.

#### 3.3.2. RF Mouse

Lung adenoma is inducible with ionizing radiation in both male and female RFM/Un mice when they are exposed at 10–12-week old [201,202]. Following a single irradiation with 9.0 Gy to the thorax roughly 87% of female RFM/Un mice develop lung cancer within 6–9 months with an average of 1.8 tumors per mouse. With a dose of 10.0 Gy 54% of male RFM/Un develop the same malignancy with a tumor multiplicity of 0.8 tumors per mouse within 11 months. However, there is a relatively high incidence of spontaneous lung carcinogenesis, at 28% in females and ~32% in males over the course of their lifespan [201,202].

#### 3.3.3. BALB/c Mouse

A single TBI dose of 2.0 Gy at 12 weeks of age with a high dose rate (0.35 Gy/min) administered to a female BALB/c/An mouse, on average, results in a 37% induction of lung adenocarcinoma. The rate of spontaneous lung adenocarcinoma is between 11–14% [203]. Fractionation at 2.0 Gy per dose does not increase the incidence of lung carcinoma when compared to a single acute exposure at low dose-rates [204].

#### 3.3.4. Lung Cancer-Associated Molecular Pathologies

The effects of the dose, dose-rate, fractionation and radiation quality on lung carcinogenesis in the mouse have been studied extensively, but the underlying molecular pathologies were more difficult to investigate until the recent emergence of genetically engineered mice (GEM). Most molecular pathologies identified in GEM are yet to be correlated with molecular pathologies observed in inbred animals [199,201,202,203,204,205]. Some data have been retroactively extrapolated from radiation studies involving 40,000 B6CF1 hybrid mice (C57BL/6 females × BALB/c male) conducted at Argonne National Laboratory between 1971 and 1986 [206,207]. Genetic material extracted from lung tissues preserved in paraffin and amplified with PCR from animals with adenocarcinomas and controls revealed that a significant percentage of animals with either radiation-induced or spontaneous lung adenocarcinoma have deletions of *Rb*, a tumor suppressor. Zhang and Woloschack reported that 97% of samples with *Rb* deletions also carried *p53* deletions and concluded that *p53* mutations may be one of the predominant mutations leading to radiation-induced lung carcinogenesis in B6CF1 mice [207]. Additionally, the same methodology has uncovered a high rate of point mutations in the *K-ras* gene in spontaneous (75%) irradiation-induced (50%) mouse lung adenocarcinomas [208].

Salient features of human lung tumors, be they carcinogen-induced or spontaneous, are also shared by murine lung cancers with alterations in *p53*, *K-ras*, and *Rb* among others [209,210,211]. *p53* is a tumor suppressor and cell cycle regulator, commonly known as the “guardian of the genome”, is either deleted or mutated in 80% of primary lung tumors [212,213,214,215]. Its loss is associated with poor clinical outcome [209]. It is thought that *p53* antitumor activity is tightly linked to apoptosis induction [216]. Retinoblastoma protein (*Rb)* and its associated pathway is another tumor suppressor mechanism that is either directly or indirectly inactivated in a variety of tumors, including a 90% rate in human small cell carcinomas [217,218]. *Rb* in involved in the regulation of cell cycle progression from G_1 _to S [219]. Proto-oncogene *K-ras,* involved in cell differentiation, growth, and apoptosis [220], is mutated in 20–30% of human lung adenocarcinomas [221]. Activated *K-ras* is also associated with poor clinical prognosis [222]. Table 5 summarizes the relevant molecular pathologies linked to IR lung cancer.

The data on the underlying molecular and pathophysiological basis of radiation-induced lung cancer in animal models is rather lacking, but nevertheless, the use of these inbred models can be valuable in testing therapeutic agents against secondary cancers in man. Further research into the mechanisms of induction and promotion of IR-induced lung carcinogenesis in inbred mice has the potential to uncover novel therapeutic targets for preventing secondary neoplastic malignancies in man following radiation therapy. Genetically engineered mice mimicking human cancers, such as *K-ras* knockout mouse model of lung adenocarcinoma [12], are very useful and sophisticated models but are potentially self-limiting and biased. These animals are predisposed to develop only a certain type of malignancy along a designated progression route and do not allow for the study of alternative mechanisms of carcinogenesis. If radiation-induced lung carcinogenesis does not follow the pre-programmed initiation and progression in a certain GEM employed, then the studies using these mice are not of general use and therapies based on these models might not be effective. Using inbred mice, however, may present a more unbiased approach to these “discovery” studies.

**Table 5:** Molecular pathologies associated with radiation-induced lung cancer.

Mouse Strain	Malignancy	Molecular Pathology	Role in Cancer	Ref.
B6CF1	Lung Adenocarcinoma	—*Rb* deletions/point mutations	Tumor suppressor; cell cycle progression control from G_1 _to S —Inactivated in 90% small cell carcinomas	[207,217,218,219]
		—*p53* deletions/point mutations	Tumor suppressor; cell cycle regulator and apoptosis inducer Deleted or mutated in 80% of primary lung tumors and other cancers	[209,212,213,214,215,216]
		—*K-ras* point mutations	Proto-oncogene; cell growth and differentiation Mutated *RAS* found in 20–30% of lung adenocarcinoma	[208,220,221,222]

### 3.4. Radiation-Induced Breast Cancer

Early compelling data linking radiation and breast cancer have been gathered from the Japanese female survivors of the atomic bomb attacks, females subjected to diagnostic fluoroscopes in Massachusetts tuberculosis sanatoria, and women treated for postpartum mastitis in New York [5]. In fact, in the Japanese atomic bomb survivors breast carcinoma presents the greatest radiation-induced increase in relative risk among all solid tumors [4]. The Massachusetts study demonstrated that females exposed to over a hundred diagnostic X-rays over the years were 80% more likely to develop breast tumors [223]. More reports are constantly emerging, implicating radiation therapy in secondary breast cancers. All of these studies demonstrate dependency on age during exposure. Up to 35% of women treated with radiation for Hodgkin’s disease at an early age developed breast cancer by the age of forty. Bhatia and Sankila studies approximated secondary radiation-induced breast cancer latency to over 10 years following radiation exposure [224,225]. Stovall and colleagues reported that an absorbed dose of >1Gy to the contralateral breast during radiotherapy is linked to a high risk of secondary *de novo* contralateral breast cancer (CBC) [226]. Risk for CBC was also linked with the reproductive history of a patient: women who have never given birth or became pregnant after the first diagnosis and subsequent radiation therapy were more likely to develop CBC than matched controls [227].

Ionizing radiation is a well-established etiological agent of both rodent and human breast cancer [190,203,228,229,230,231,232,233]. Despite the fact that mammary cancer mouse models are somewhat dissimilar from human breast cancers—low frequency of hormonal dependence of the tumor and carcinomas originating in the alveolar tissue—they are nonetheless valuable in studying chemotherapeutic preventative and therapeutic agents in addition to modeling the underlying molecular pathology [230]. The BALB/c mouse has been used extensively as a model of mammary cancer either with cancer either being induced with a TBI or through the implantation of irradiated tissues into syngenic mice [234]. Table 6 summarizes the most commonly used BALB/c models.

**Table 6:** Induction of breast cancer in mice with low-LET ionizing radiation.

Malignancy	Mouse Strain	Age	Sex	Mode of Induction	Latency	Spontaneous Frequency	Induced Frequency	Ref.
Breast Cancer	BALB/c	12 weeks	Female	2.0 Gy exposure (TBI)	~24 months	8%	22%	[235]
Breast Cancer	BALB/c orthograft	12 weeks	Female	1.0 Gy TBI of donor cells	10 weeks	<1%	* Dysplasia ~75% * Tumors ~25%	[236]
Breast Cancer	BALB/c chimera	12 weeks	Female	4.0 Gy TBI of host	6 weeks	~19%	~81%	[237,238,239,240]

* Dependent on the passage status of the donor cells.

#### 3.4.1. BALB/c Whole-Body Exposure Model

Original studies on BALB/c females whole-body irradiated with gamma rays have shown an increase in mammary carcinogenesis, from a background frequency of around 8% to about 22% within the mouse’s lifetime. To induce mammary adenocarcinoma, BALB/c females are irradiated at 12-weeks of age with a total dose of 2.0 Gy at a relatively high dose-rate of ~0.35 Gy/min; irradiation with the same total dose but a much smaller dose-rate of 0.083 Gy/day results in roughly half of the high dose-rate frequency at ~13% [203]. In fact, even a dose of 0.25 Gy but at the high dose-rate of 0.35 Gy/min induces mammary tumors in roughly 20% of animals [204]. Irradiation does not change latency but rather affects the incidence of breast adenocarcinomas. Prior to the appearance of tumors, hyperplastic lesions in the ductal dysplasia are detected 12–14 months after IR exposure [235]. Sensitivity to radiation-induced breast adenocarcinoma in the BALB/c female has been attributed to polymorphisms of *Prkdc*, a DNA-dependent protein kinase involved in DNA repair and post-IR cell signaling [241]. This model, however, is plagued by ovarian tumors detected in over 90% of autopsied mice [203].

#### 3.4.2. BALB/c Syngenic Transplant Model

A great leap forward in the field of breast cancer biology was made in 1959 when DeOme and colleagues introduced a murine orthograft model of breast cancer. The model involves clearing of the fat pad in 3-week-old female virgin mice, followed by a transplant of a 1mm duct fragment from a donor mouse containing hyperplastic lesions [236,242]. Ethier and Ullrich successfully adapted this model from the original C3H mice to BALB/c and later extensively used it to demonstrate strain sensitivity differences and the associated molecular mechanism [236,243,244,245]. Additionally, Barcellos-Hoff and colleagues employed this model to further revolutionize the cancer research field demonstrating the importance of tissue microenvironment in breast carcinogenesis [237,238,239,240].

In the “cell dissociation assay” or the *in vitro*/*in vivo* model employed by Ethier and Ullrich, virgin donor BALB/c females are TBI irradiated with a total dose of 1.0 Gy at 12-weeks of age, then their mammary tissues are removed at 24 hours post-exposure. Single-cell suspensions of 10^4^ cells, prepared from these donor animals, are then injected into 3-week-old virgin BALB/c females whose mammary fat pad had been removed. At 10 weeks following the procedure, the mice are sacrificed and the outgrowths are removed and analyzed for pathologies in the ductal architecture. Normal outgrowths have 2–3 terminal ducts that are capped by end buds in the fat pad and resemble anatomically correct ducts. Abnormal outgrowths have up to 10 or more terminal ducts capped with hyperplastic end buds are and assigned an arbitrary classification I–III, with Class III being the most severe [244,246,247].

In a series of elegant experiments, Ullrich and colleagues demonstrated thaT cells from the irradiated donor harvested at different time points after irradiation, passaged *in vitro,* and transplanted into unirradiated recipient mice develop either dysplasia or adenocarcinomas depending on the time of harvesting or the number on passages in culture prior to implantation. Cells harvested at 52 weeks post-IR and injected into recipient host tend to regenerate dysplastic outgrowths at a high rate (3 in 4) and develop into tumors (1 in 4) while cells harvested at 1–16 weeks develop into normal outgrowths unless they have undergone extensive *in vitro* passaging. Dysplasia and tumors resembled *in situ* tumorigenesis with leukocyte infiltrations and angiogenesis [235].

Barcellos-Hoff and Ravi capitalized on this model and have established a radiation chimera model of their own [248] in which the fat pads of BALB/c mouse hosts are cleared at 3-weeks of age and the mice themselves are TBI irradiated with 4.0 Gy at 10–12-weeks of age. Three days later these hosts are subsequently transplanted with immortalized but non-malignant, unirradiated COMMA-D mouse epithelial cells from midpregnant BALB/c females [249]. At 6 weeks post-IR, the cells injected into irradiated host have 81% tumor penetrance as compared to 19% in the unirradiated host. Alternatively, a 1 mm^3^ fragment of the formed epithelia from a wildtype donor or a donor primed for neoplastic development can be transplanted into the irradiated host whose mammary fat pads have been cleared [250]. This syngenic mode demonstrates that radiation-induced changes in the stromal microenvironment contribute to carcinogenicity [248].

#### 3.4.3. Breast Cancer-Associated Molecular Pathologies

Cell lines established from female BALB/c donors irradiated with 1.0 Gy and harvested at 4 weeks (EF42) or 16 weeks (EF137) were used in early studies to examine molecular pathologies leading to tumorigenesis. Reduced or absent *Rb* protein was detected in EF42 after 11 passages and in EF137 after only 6 passages in culture. Mutant *p53* was detected in 95% after >20 passage and 1–5% in passages 6–10 suggesting that it is an early transformation event in preneoplastic cells. Additionally, following 20 passages in culture angiogenesis is often detected [235]. Ethier and Ullrich reported that injecting a larger number of cells results in a less frequent and less pronounced dysplasia as compared to an injection with fewer cells [244,246]. This observation suggests that replicative stress might be contributing to a faster and more prominent progression to ductal dysplasia as in the case with TL.

Barcellos-Hoff and colleagues have linked rapid remodeling of the microenvironment observed in the irradiated mammary gland to changes in the extracellular matrix and latent Transforming Growth Factor Beta (TGF-β) expression [237,251,252,253] and later showed that it accelerates tumor progression [250]. TGF-β is involved in regulating a variety of cell processes including cell cycle control, apoptosis and cell differentiation among others [252,254]. Radiation-induced activation of TGF-β has been additionally implicated in cell fate decisions and influence DNA-repair kinetics in an ATM-dependent manner [255,256].

The radiation chimera model is able to capture salient features of breast cancer that are thought to arise after irradiation, despite the fact that, unlike in the case of human malignancy, the transplanted epithelium itself has not been irradiated. Human breast cancer associated with radiation exposure has been shown to initiate in the ducT cells that often infiltrate the rest of the breast tissue [5] similarly to the way mammary cancer arises in the transplantation models. Barcellos-Hoff and colleagues have also reported that tumors arising from transplanted epithelium lack functional *p53* protein and are estrogen receptor (ER) negative [250], akin to those observed in women with breast cancer who have been previously irradiated [257]. *Rb* deficiencies observed by Ullrich and Preston in neoplastic ducT cells have also been reported in human breast cancer tumors and correlated with a highly invasive tumor phenotype [258]. Table 7 summarizes relevant molecular pathologies in radiation-induced breast cancer studies. 

**Table 7:** Molecular pathologies associated with radiation-induced breast cancer.

MouseStrain	Malignancy	Molecular Pathology	Role in Cancer	Ref.
BALB/c	Mammary Adenocarcinoma	—Reduction or loss of *Rb*	Tumor suppressor; cell cycle progression control from G_1 _to S Inactivated in 90% small cell carcinomas	[235,258]
		—*p53* mutation	Tumor suppressor; cell cycle regulator and apoptosis inducer Among the key mutations in breast cancer initiation	[235,250,257]
		—TGF-β expression	Cell cycle control; apoptosis; cell differentiation –Linked to pro-tumorigenic microenvironment	[245,251,252,253,254,255,256]

## 4. Conclusions

An ideal mouse model of radiation-induced carcinogenesis would have a low spontaneous background frequency of the desired malignancy, would not co-develop cancers at alternative sites, would have a short latency period and would have tumors that are nearly identical to corresponding human disease in their onset, progression and underlying pathology. Such a perfect model, however, does not exist and we are therefore forced to compromise on some of these features. While we can, perhaps, compromise on the latency of the cancers and the frequencies of inductions, we cannot afford to compromise on the molecular and pathophysiological similarities to human radiation-induced malignancies that these models must mimic. Much work in the field of radiation oncology remains to be done in order to develop more accurate recapitulations of human radiation-induced cancers. Today we are still at the stage where we still have difficulty discerning radiation-induced secondary cancers from primary tumors in men because the molecular signatures of each type remain to be established. Relating these molecular signatures to tumors that arise in mice following IR is yet another degree of difficulty.

Murine models presented within the scope of this review are most often a compromise on background frequencies and rates of induction, but they do demonstrate strong molecular and phenotypic correlations to salient features of the human cancers they are meant to represent. This enables these models to be rightfully employed to test the extent of therapeutic benefits of candidate drugs against radiation-induced carcinogenesis.
